# Apoptosis in the trabecular meshwork of glaucomatous patients

**Published:** 2008-08-18

**Authors:** Jimena Baleriola, Julián García-Feijoo, José M. Martínez-de-la-Casa, Arturo Fernández-Cruz, Enrique J. de la Rosa, Raquel Fernández-Durango

**Affiliations:** 1 3D Lab (Development, Differentiation, & Degeneration), Department of Cellular and Molecular Physiopathology, Centro de Investigaciones Biológicas, CSIC, Madrid, Spain; 2 Department of Ophthalmology, Hospital Clínico San Carlos, Madrid, Spain; 3 Research Unit, Department Internal Medicine III, Hospital Clínico San Carlos, Madrid, Spain

## Abstract

We established and validated an in toto method to perform TdT-mediated dUTP nick end labeling to study apoptosis in human trabecular meshwork tissue obtained during trabeculectomy in glaucoma patients. In specimens from patients with primary open-angle glaucoma and primary angle-closure glaucoma, we detected a tendency for more apoptotic cells to accumulate in patients with primary open-angle glaucoma. The utility of this method to study apoptosis in the trabecular meshwork is discussed, as well as its application as a tool in biologic samples.

## Introduction

Glaucoma is one of the most common causes of blindness in the world and it is a progressive optic neuropathy that provokes the loss of vision [1]. Glaucoma is characterized by retinal ganglion cell death and cupping of the optic disc. Primary open-angle glaucoma (POAG) is the most common form of the disease and it is closely associated with an increase in intraocular pressure (IOP) that results from an excessive resistance to the outflow of the aqueous humor (AH) through the conventional pathway [2]. This pathway involves both the trabecular meshwork (TM) and Schlemm’s canal (SC); the latter is responsible for the outflow of AH from the anterior chamber to the venous system and it is situated at the angle formed by the cornea and iris [3]. In the TM, arrays of collagen beams are covered by endothelium-like cells and the space between the beams is occupied by extracellular matrix (ECM). The adjacent SC is a continuous endothelium-lined channel that drains off AH into the bloodstream. The TM is anchored by tendons of the longitudinal ciliary muscle (CM) and by elastin fibers that connect it to the endothelium of the SC. Both the TM and the longitudinal CM are actively involved in regulating AH outflow and IOP. Indeed, these two structures act as functional antagonists, since contraction of the CM leads to a distension of the TM and the ensuing reduction in outflow, whereas contraction of the TM produces the opposite effect [4]. It is thought that the resistance to aqueous outflow occurs at the junction where the TM meets the inner wall of the SC, the juxtacanalicular region. Thus, the TM represents the key region in the pathogenesis of glaucoma and it is a good potential target for therapeutic interventions.

The most characteristic structural alterations in the TM of eyes with POAG involve the decrease in cellularity [5] and an increase in the ECM, as well as the presence of “plaque material” in the juxtacanalicular tissue. This “plaque material” is an accumulation of banded fibrillar elements in which different glycoproteins are embedded, and it derives from thickened sheaths of elastic fibers [6]. These alterations in the TM may produce critical changes in outflow resistance.

The loss of cells within the TM is more severe in POAG patients than in normal age matched controls [5] and it is thought to be an early event in POAG [7] whereby the TM endothelial cell population diminishes with age [7,8]. At 20 years of age the entire meshwork has been estimated to contain 763,000 cells and this number decreases to 403,000 by the age of 80, with a loss of approximately 6,000 cells per year [8]. The exact mechanism by which the cell population is reduced in normal and glaucomatous human TM tissues is not known, although several potential mechanisms have been suggested, including wear-and tear, phagocytosis, cell migration, and cell death [9]. Cell death may occur in different ways, including apoptosis (type I), autophagy (type II), and necrosis (type III), each of which is related with a particular series of events [10]. Significantly, human TM cell lines, and ex vivo dissected TM tissue obtained from normal donors, express several modulators of apoptosis (Fas, Bcl-2, Bcl-Xl, Bax, and caspases), and in addition, human TM cells can be stimulated to undergo apoptosis via the Fas-FasL pathway [11]. However, to our knowledge apoptosis has not been detected in the TM from glaucomatous patients.

DNA fragmentation is a typical feature of apoptotic cell death and this process can be assessed via the activity of multiple DNases, including DNase I that cleaves DNA leaving 3′-OH DNA ends. During apoptosis, caspase-activated DNase (CAD) digests genomic DNA into oligonucleosomal fragments that are further cleaved by DNase I [12]. Thus, terminal deoxynucleotidyl transferase (TdT)-mediated dUTP nick end labeling (TUNEL) can take advantage of the increase in free 3′-OH ends. The TdT enzyme can incorporate fluorescein isothiocyanate (FITC) conjugated dUTP nucleotides into these fragments, thus enabling the cells with fragmented DNA to be visualized by fluorescence microscopy. By applying TUNEL to whole specimens it is possible to define rare cell death events, as shown previously during early retinal development [13]. We established such a method to evaluate whether or not apoptotic cell death might contribute to the loss of cells within the TM. We successfully identified apoptotic cells in human TM specimens obtained during trabeculectomy. We examined patients with POAG as well as those with PACG (primary angle-closure glaucoma), the latter characterized by an obstruction to the outflow of AH that produces an increase in IOP due to the anatomic predisposition of the eye.

## Methods

### Study population

Human tissue was handled in accordance with the Helsinki Declaration and the Local Committee of Ethics for experimentation with human tissues. Institutional approval was obtained and all the patients enrolled in the study provided their informed voluntary written consent. Seven patients with POAG and four with PACG were included in the study, in which only one eye was studied. The age of the patients ranged from 55 to 85 years (mean age±SD, 71±12 years) and the disease duration ranged from 4 to 12 years (Table 1 summarizes their demographic characteristics). The patients were prospectively selected based on the following criteria. The inclusion criteria were: glaucoma (POAG or PACG), more than 50 years of age, access to at least three reliable baseline preoperative visual fields, no retinal or neurologic disease that may have affected the visual field, and being treated with the same combination of drugs such as a β-blocker (timolol maleate), a carbonic anhydrase inhibitor (dorzolamide) and an α_2_ adrenergic agonist (brimonidine). The exclusion criteria were: ocular disease other than glaucoma, normal tension glaucoma, pseudoexfoliation or pigmentary syndrome, previous eye surgery or laser trabeculoplasty (argon laser trabeculoplasty or selective laser trabeculoplasty), diabetes mellitus, uveitis, systemic collagenopathy, and objective neurologic signs.

**Table 1:** Demographic characteristics of the patients enrolled in the study.

**Statistics**	**POAG**	**PACG**
Number	7	4
mean age years (±SD)	78±8	71±12
IOP (mm Hg; mean±SD)	24.2±2.1	22.5±4

Abbreviations in the table are: POAG, primary open-angle glaucoma; PACG, primary angle-closure glaucoma; and IOP, intraocular pressure.

POAG was defined as the presence of a reproducible visual field defect consistent with glaucoma and the appearance of the optic disc, coupled with a pretreatment IOP of at least 21 mmHg and an open angle with no signs of secondary causes of glaucoma. The PACG diagnosis was based on the same criteria except that the angle was occludable before laser iridotomy with the posterior TM visible for less than 90° of the angle circumference. IOP was determined using the Goldmann applanation tonometer (GAT; Haag-Streit, Koeniz, Switzerland).

### Stratification of patients

Perimetry was performed during the four week period before surgery (Octopus tG1; Interzeag AG, Switzerland). We divided the patients into four groups based on Mean Defect (MD) values: MD greater than −6 dB (mild visual field loss), MD between −6 and −12 dB (moderate visual field loss), MD between −13 dB and −20 dB (severe visual field loss), and MD greater than −20 dB (terminal).

### Collection of trabecular meshwork specimens

The experimental protocol required the removal of TM specimens during trabeculectomy. The surgical technique employed has been described elsewhere [14], and it involved the use of a large flap (scleral flap size: 8–10x5–6 mm, flap thickness: 2/3 scleral thickness; excised inner block size: 6–5x4 mm) that enabled us to obtain a large specimen. The TM specimens were obtained according to standard surgical procedures and for all specimens, a 45° knife was used to cut a 6–5x4 mm button of corneoscleral tissue. The TM was then dissected out under a microscope, and the TM biopsies were snap-frozen in liquid nitrogen and stored at −80 °C until they were assayed.

### Detection of apoptosis

TUNEL was performed on whole TM specimens to label fragmented DNA with FITC-dUTP based on previous methods [13,15]. Briefly, frozen samples were thawed on ice and fixed overnight in 4% paraformaldehyde (w/v, Sigma, St. Louis, MO) prepared in 0.1 M phosphate buffer (PB, pH 7.4). The TM tissue was permeabilised for 2 h at RT (changing the solution every 30 min) with 4% Triton X-100 in PBS (w/v, Fluka, Buchs, Switzerland), it was mildly digested for 15 min at 37 °C with 20 μg/ml proteinase K in PBS (Promega, Madison, WI), and it was then processed for TUNEL following the manufacturer’s instructions (Apoptosis Detection System, Promega). At the end of the assay, the TM tissue was counterstained with 4',6-diamidino-2-phenylindole (DAPI), mounted with Vectashield mounting medium (Vector Laboratories, Burlingame, CA) and visualized on a confocal microscope (LEICA DMRE2, Heidelberg, Germany). The number of apoptotic cells was determined by counting TUNEL positive cells on images representing the maximal projection of confocal series under 10X and 20X objectives.

## Results

### Programmed cell death can be detected in the TM of glaucomatous patients

**Figure 1:**
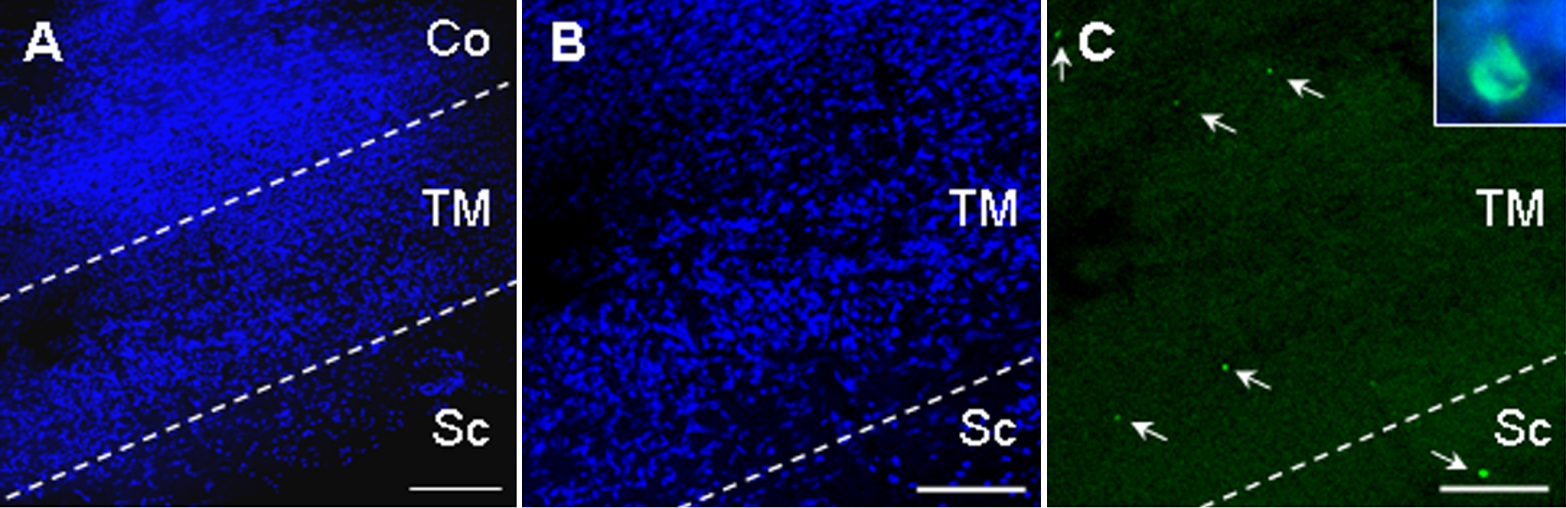
TUNEL positive cells can be detected in TM samples from glaucomatous patients. TM samples were stained with DAPI (**A**,**B**) and by TUNEL (**C**). The DAPI staining allowed us to visualize the structure of the specimen and to identify apoptotic nuclei both within and outside of the TM. Dotted lines represent the limits of the TM and the arrows indicate the presence of apoptotic cells in the TM and sclera of a POAG patient. The inset in **C** shows a pyknotic nucleus (visualized by DAPI blue staining) that is also positive for TUNEL (green). Abbreviations in the figure are Co, Cornea; TM, Trabecular Meshwork; Sc, Sclera. The scale bar is equal to 250 μm in **A** and 150 μm in **B** and **C**.

TM samples processed for TUNEL were counterstained with DAPI to visualize the limit of the TM, as well as the corneal and scleral surfaces (Figure 1A,B). In addition, DAPI staining also enabled pyknotic and condensed nuclei to be identified, morphological features of programmed cell death. We found TUNEL positive cells in the sclera (Figure 1C) and the cornea (data not shown) of all specimens analyzed, indicating that programmed cell death occurred in this tissue as described previously [16]. Indeed, in 8 of the 11 samples analyzed (72.7%) there were TUNEL stained cells in the TM (Figure 1C). To ensure that the green TUNEL staining corresponded to apoptotic nuclei we captured the same confocal images in the green (TUNEL) and blue (DAPI) channels (box in Figure 1C), confirming that the TUNEL cells corresponded to those with pyknotic nuclei.

### There is more cell death in the trabecular meshwork from POAG patients than in that of PACG patients

**Figure 2:**
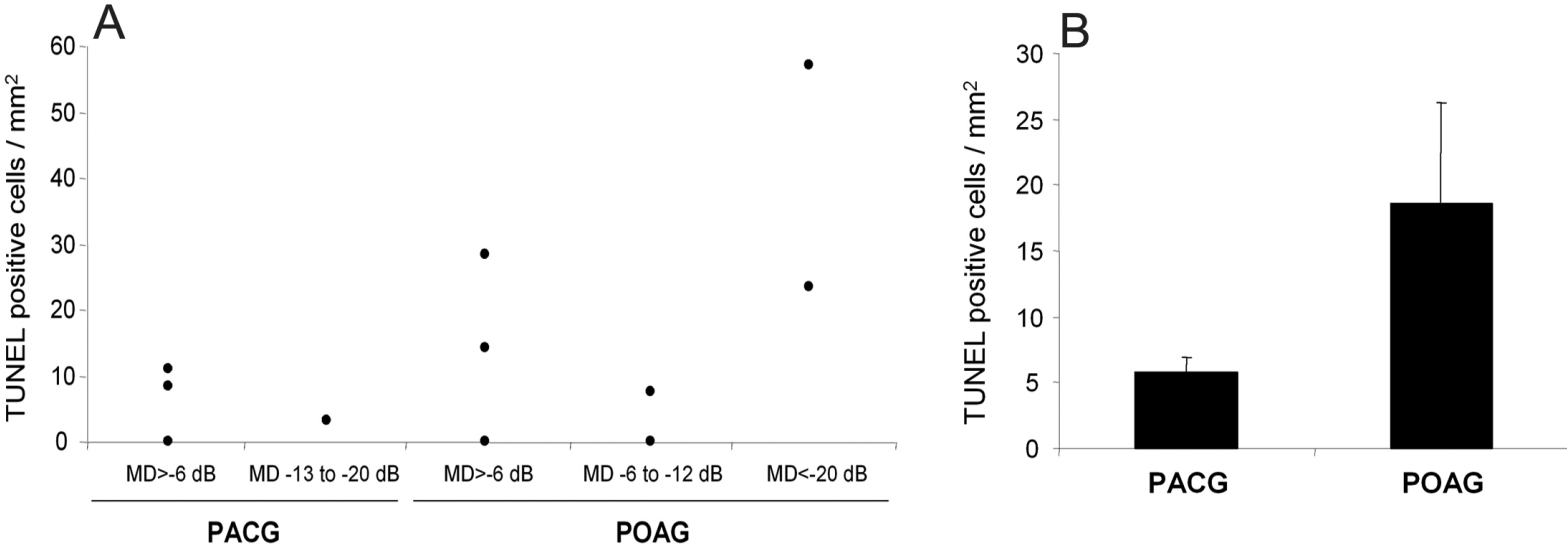
Scoring of TUNEL positive cells in the TM of POAG and PACG patients. TUNEL-positive apoptotic cells were scored in maximal projection images of confocal series taken under 10X and 20X objectives and represented as TUNEL-positive cells per area. **A**: The values are represented individually and classified according to the Mean Defect (MD) criterion and the type of glaucoma (PACG or POAG). **B**: Values (mean±SD) grouped for PACG or POAG patients.

We successfully identified apoptotic cells in human TM specimens obtained during trabeculectomy. Apoptotic cells were scored in images representing the maximal projections of the confocal series throughout the whole TM. Figure 2A represents TUNEL positive cells identified in the TM of each POAG and PACG patients against their correspondent visual fields, expressed as MD values. The accumulated numbers showed a clear tendency toward an increase in the number of TUNEL positive cells in specimens collected from POAG patients (Figure 2B) indicating a greater incidence of apoptosis associated with this condition.

## Discussion

In this communication, we demonstrate that programmed cell death occurs in the TM of POAG and PACG patients, although the number of apoptotic cells may be higher in the former condition. These observations were achieved by developing a TUNEL method to stain the TM as a whole, facilitating the visualization and scoring of the relatively low number of apoptotic cells in this tissue.

Our observations may explain the loss of cells described previously in POAG patients [5,7] and they suggest that apoptosis could be one of the mechanisms by which trabecular cells die in glaucoma. The differences observed in the number of apoptotic cells between POAG and PACG patients are unlikely to be due to the antiglaucoma treatment since these patients all received the same type of anti-glaucoma medication: β-blocker (timolol), carbonic anhydrase inhibitor (dorzolamide) and α_2_ adrenergic agonist (brimonidine). While it is possible that the antiglaucoma treatment may have induced apoptosis in the TM of glaucomatous patients, exposing cultured TM to anti-glaucoma drugs such as timolol and pilocarpine does not induce apoptosis [17]. Moreover, the effect of benzalkonium-preserved (BAC+) or preservative-free (BAC-) antiglaucoma medication on the expression of apoptotic markers in cultured human TM cells has also been evaluated. At concentrations higher than those thought to be found in the AH after instillation, unpreserved beta-blockers (timolol and betaxolol) and prostaglandin (latanoprost) did not exhibit any pro-apoptotic activity on TM cells in vitro [18]. However, we cannot rule out that the long-term administration of antiglaucoma treatment might contribute to the apoptosis of TM cells.

The relationship between TM cell loss and the apoptosis observed in POAG patients deserves further characterization, as do the mechanisms underlying the apoptosis in the TM. It has been suggested that intense phagocytic activity of trabecular cells could lead to cell death [19]. Furthermore, glaucoma itself could also produce apoptosis of TM cells through mechanical stress [20] or through trabecular hypoperfusion [21]. An increase in oxidative stress may also contribute to cell loss or alterations in the functioning of TM cells [22,23].

Finally, we have to mention two limitations of this study. One is the absence of the control group since it is impossible to obtain TM specimens from control subjects. The other one is the small number of subjects included in each group due to the difficulty of obtaining TM samples from glaucoma patients which fulfill the strict inclusion and exclusion criteria. Despite these restrictions the technique is still suitable to study cell death in the TM and will enable us to more accurately evaluate the different alternatives for the etiology of the pathological stage, as well as of the effectivity of certain therapeutic approaches. This method may also be applicable to study apoptosis in other human tissues obtained by surgery.

## References

[1] Quigley HA, Broman AT (2006). The number of people with glaucoma worldwide in 2010 and 2020.. Br J Ophthalmol.

[2] Quigley HA (1993). Open-angle glaucoma.. N Engl J Med.

[3] Bill A, Phillips CI (1971). Uveoscleral drainage of aqueous humor in human eyes.. Exp Eye Res.

[4] Wiederholt M, Thieme H, Stumpff F (2000). The regulation of trabecular meshwork and ciliary muscle contractility.. Prog Retin Eye Res.

[5] Alvarado J, Murphy C, Juster R (1984). Trabecular meshwork cellularity in primary open-angle glaucoma and nonglaucomatous normals.. Ophthalmology.

[6] Lutjen-Drecoll E, Shimiau T, Rohrbach M, Rohen JW (1986). Quantitative analysis of 'plaque material' in the inner- and outer wall of Schlemm's canal in normal- and glaucomatous eyes.. Exp Eye Res.

[7] Alvarado J, Murphy C, Polansky J, Juster R (1981). Age-related changes in trabecular meshwork cellularity.. Invest Ophthalmol Vis Sci.

[8] Grierson I, Howes RC (1987). Age-related depletion of the cell population in the human trabecular meshwork.. Eye.

[9] Grierson I, Hogg P (1995). The proliferative and migratory activities of trabecular meshwork cells.. Prog Retin Eye Res.

[10] Krysko DV, Van den Berghe T, D'Herde K, Vandenabeele P (2008). Apoptosis and necrosis: detection, discrimination and phagocytosis.. Methods.

[11] Agarwal R, Talati M, Lambert W, Clark AF, Wilson SE, Agarwal N, Wordinger RJ (1999). Fas-activated apoptosis and apoptosis mediators in human trabecular meshwork cells.. Exp Eye Res.

[12] Mukae N, Enari M, Sakahira H, Fukuda Y, Inazawa J, Toh H, Nagata S (1998). Molecular cloning and characterization of human caspase activated DNase.. Proc Natl Acad Sci USA.

[13] Díaz B, Pimentel B, de Pablo F, de la Rosa EJ (1999). Apoptotic cell death of proliferating neuroepithelial cells in the embryonic retina is prevented by insulin.. Eur J Neurosci.

[14] Lázaro C, García Feijoo J, Castillo A, Perea J, Martínez-Casa JM, García-Sanchez JM (2007). Impact of intraocular pressure after filtration surgery on visual field progression in primary open-angle glaucoma.. Eur J Ophthalmol.

[15] Mayordomo R, Valenciano AI, de la Rosa EJ, Hallbook F (2003). Generation of retinal ganglion cells is modulated by caspase-dependent programmed cell death.. Eur J Neurosci.

[16] Yew DT, Sha O, Li WW, Lam TT, Lorke DE (2001). Proliferation and apoptosis in the epithelium of the developing human cornea and conjunctiva.. Life Sci.

[17] Sibayan SA, Latina MA, Sherwood ME, Flotte TJ, White K (1988). Apoptosis and morphological changes in drug-treated trabecular meschwork cells in vitro.. Exp Eye Res.

[18] Hamard P, Blondin C, Debbasch C, Warnet JM, Baudouin C, Brignole F (2003). In vitro effects of preserved and unpreserved antiglaucoma drugs on apoptotic marker expression by human trabecular cells.. Graefes Arch Clin Exp Ophthalmol.

[19] Johnson DH, Richardson TM, Epstein DL (1989). Trabecular meshwork recovery after phagocytic challenge.. Curr Eye Res.

[20] Grierson I (1987). What is open angle glaucoma?. Eye.

[21] Rohen JW, Lutjen-Drecoll E, Flugel C, Meyer M, Grierson I (1993). Ultrastructure of the trabecular meshwork in untreated cases of primary open-angle glaucoma.. Exp Eye Res.

[22] De La Paz MA, Epstein DL (1996). Effect of age on superoxide dismutase activity of human trabecular meshwork.. Invest Ophthalmol Vis Sci.

[23] Fernández-Durango R, Fernández-Martínez A, García-Feijoo J, Castillo A, de la Casa JM, García-Bueno B, Pérez-Nievas BG, Fernández-Cruz A, Leza JC (2008). Expression of nitrotyrosine and oxidative consequences in the trabecular meshwork of patients with primary open-angle glaucoma.. Invest Ophthalmol Vis Sci.

